# BRCA1 and BRCA2 tumor suppressors in neural crest cells are essential for craniofacial bone development

**DOI:** 10.1371/journal.pgen.1007340

**Published:** 2018-05-02

**Authors:** Kohei Kitami, Megumi Kitami, Masaru Kaku, Bin Wang, Yoshihiro Komatsu

**Affiliations:** 1 Department of Pediatrics, The University of Texas Medical School at Houston, Houston, TX, United States of America; 2 Division of Orthodontics, Niigata University Graduate School of Medical and Dental Sciences, Niigata, Japan; 3 Division of Bioprosthodontics, Niigata University Graduate School of Medical and Dental Sciences, Niigata, Japan; 4 Department of Genetics, The University of Texas MD Anderson Cancer Center, Houston, TX, United States of America; 5 Graduate Program in Genes and Development, The University of Texas Graduate School of Biomedical Sciences at Houston, Houston, TX, United States of America; Stowers Institute for Medical Research, UNITED STATES

## Abstract

Craniofacial abnormalities, including facial skeletal defects, comprise approximately one-third of all birth defects in humans. Since most bones in the face derive from cranial neural crest cells (CNCCs), which are multipotent stem cells, craniofacial bone disorders are largely attributed to defects in CNCCs. However, it remains unclear how the niche of CNCCs is coordinated by multiple gene regulatory networks essential for craniofacial bone development. Here we report that tumor suppressors breast cancer 1 (BRCA1) and breast cancer 2 (BRCA2) are required for craniofacial bone development in mice. Disruption of *Brca1* in CNCC-derived mesenchymal cells, but not in epithelial-derived cells, resulted in craniofacial skeletal defects. Whereas osteogenic differentiation was normal, both osteogenic proliferation and survival were severely attenuated in *Brca1* mutants. *Brca1*-deficient craniofacial skeletogenic precursors displayed increased DNA damage and enhanced cell apoptosis. Importantly, the craniofacial skeletal defects were sufficiently rescued by superimposing p53 null alleles in a neural crest-specific manner *in vivo*, indicating that BRCA1 deficiency induced DNA damage, cell apoptosis, and that the pathogenesis of craniofacial bone defects can be compensated by inactivation of p53. Mice lacking *Brca2* in CNCCs, but not in epithelial-derived cells, also displayed abnormalities resembling the craniofacial skeletal malformations observed in *Brca1* mutants. Our data shed light on the importance of BRCA1/BRCA2 function in CNCCs during craniofacial skeletal formation.

## Author summary

Craniofacial abnormalities, including facial skeletal defects, comprise approximately one-third of all birth defects in humans. Since most bones in the face derive from neural crest cells, which are multipotent stem cells, craniofacial bone disorders are largely attributed to defects in neural crest cells. However, it remains unclear how the niche of neural crest cells is coordinated by multiple gene regulatory networks essential for craniofacial bone development. Here, we show that tumor suppressor breast cancer 1 (BRCA1) and breast cancer 2 (BRCA2) are required for craniofacial bone development in mice. Our data shed light on the importance of the DNA damage response/repair machinery in neural crest cells via BRCA1/BRCA2, providing novel insights into the mechanisms of craniofacial bone development.

## Introduction

Abnormal growth of the facial bones causes congenital craniofacial malformations [1]. Because most bones in the face derive from multipotent stem cells designated cranial neural crest cells (CNCCs) [2, 3], craniofacial bone disorders are largely attributed to defects in CNCCs [4, 5]. Importantly, several bone-related diseases affect craniofacial bones, but not other bones, despite the fact that the genes involved in these diseases are expressed throughout the body [6]. This suggests that CNCCs have distinct mechanisms to regulate craniofacial bone development. Therefore, clarifying the role of CNCCs in craniofacial bone development is essential for understanding the etiology of facial bone anomalies.

Prior studies have shown that mutations in several DNA damage response proteins lead to defects in craniofacial skeletal development [7, 8]. For example, patients with Nijmegen breakage syndrome, which is caused by a mutation in NBS1, a molecule critical for the initial processing of DNA strand breaks, display small lower jaws [9]. Also, Fanconi anemia, a disease frequently associated with congenital craniofacial bone anomalies, results from a genetic defect in a cluster of genes responsible for DNA repair [10, 11]. However, it remains elusive whether DNA damage repair mechanisms in CNCCs contribute to normal craniofacial bone formation.

The tumor suppressor genes breast cancer 1 (BRCA1) and breast cancer 2 (BRCA2) are key players in DNA damage response and homologous recombination (HR), which are critical for the repair of DNA double-strand breaks to maintain the fidelity of the genome. Mutations in BRCA1/BRCA2 lead to genetic instability and frequently cause familial breast and ovarian cancers [12]. While little is known about how these tumor suppressor genes affect craniofacial development, a recent study has shown that non-syndromic cleft lip and palate, one of the most common human craniofacial deformities, are associated with dysregulation of the gene regulatory network via BRCA1 [13]. Since germline mutations in DNA repair genes have been linked to congenital defects [8], it is tempting to hypothesize that the BRCA1/BRCA2-dependent DNA damage and repair machinery is critical for the prevention of craniofacial abnormalities. Therefore, it is important to understand how BRCA1/BRCA2 function in CNCCs during craniofacial development.

The purpose of this study is to investigate the role of BRCA1 and BRCA2 in the multipotent cell population of CNCCs. We found that BRCA1 and BRCA2 are required for skeletogenic cell proliferation and survival in CNCC-derived bones, highlighting the essentiality of the BRCA1 and BRCA2 for craniofacial skeletal development.

## Results

### Disruption of *Brca1* in neural crest cells leads to craniofacial malformations in mice

**Fig 1:**
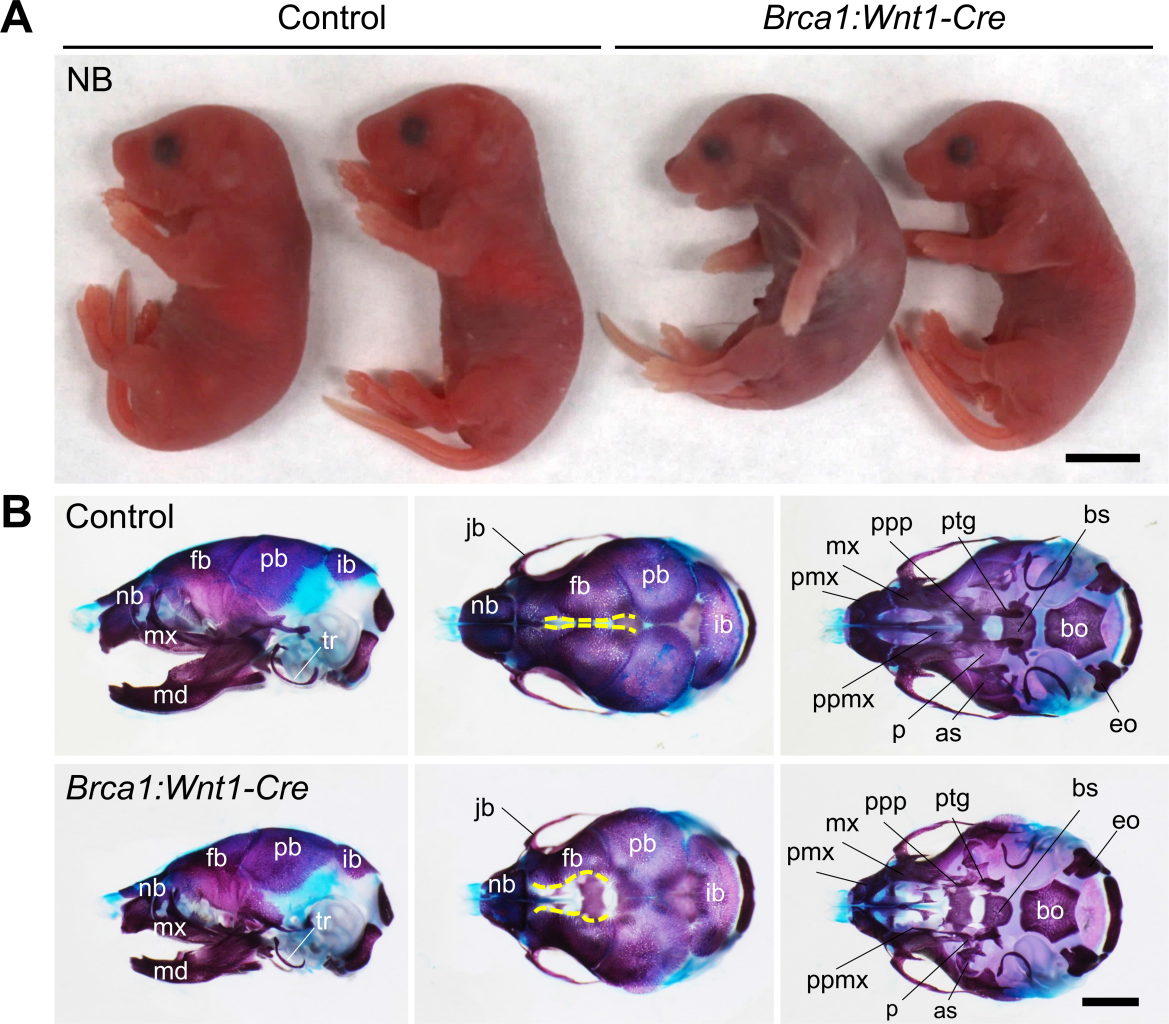
Neural crest cell-specific *Brca1* deletion causes craniofacial bone abnormalities in mice. (A) Lateral views of control and *Brca1*:*Wnt1-Cre* mice at birth (NB). Scale bar = 5mm. (B) Alcian blue- and alizarin red-stained skulls of control and *Brca1*:*Wnt1-Cre* mice at birth. Yellow broken lines indicate osteogenic fronts. Note that the frontal bones of *Brca1*:*Wnt1-Cre* mice are separated by a large open space. Scale bar = 2mm. as, alisphenoid; bo, basioccipital; bs, basisphenoid; eo, exoccipital; fb, frontal bone; ib, interparietal bone; jb, jugal bone; md, mandible; mx, maxilla; nb, nasal bone; p, palatine; pb, parietal bone; pmx, premaxilla; ppmx, palatal process of maxilla; ppp, palatal process of palatine; ptg, pterygoid; tr, tympanic ring.

To characterize the function of BRCA1 in craniofacial development, we disrupted *Brca1* in a neural crest-specific manner using a wingless-related MMTV integration site1 (*Wnt1*)-Cre driver line [14, 15]. Mice lacking *Brca1* in neural crests showed craniofacial abnormalities (*Brca1*:*Wnt1-Cre* hereafter) (Fig 1A). While *Brca1*:*Wnt1-Cre* mice were born at Mendelian ratios (S1 Table), they could not survive more than twenty-four hours, most likely due to the cleft palate phenotype (data to be published elsewhere). *Brca1*:*Wnt1-Cre* mice displayed small heads with 100% phenotypic penetrance (Fig 1A). Skeletal staining revealed that the CNCC-derived facial bones were hypoplastic in *Brca1*:*Wnt1-Cre* embryos (Fig 1B). The size of the maxillary and mandibular bones was small, and the nasal-frontal bones and membranous bones on the skull base were defective in *Brca1*:*Wnt1-Cre* mice. These results thus suggest that BRCA1 is responsible for orchestrating the formation of CNCC-derived facial bones.

Because epithelial-mesenchymal interactions are also critical for craniofacial morphogenesis [1], we disrupted *Brca1* in an epithelial cell-specific manner using a Keratin14 (*K14*)-Cre driver line [16]. The epithelial cell-specific deletion of *Brca1* did not result in any overt craniofacial abnormality (S1 Fig). Together, these results indicate that BRCA1 is indispensable for facial development in ectomesenchymal cells, but not in epithelial cells. In this study, we further examined the function of BRCA1 in craniofacial bones, especially focusing on CNCC-derived skull bones.

### *Brca1* is required for osteogenic proliferation and survival in the frontal bone primordium

**Fig 2:**
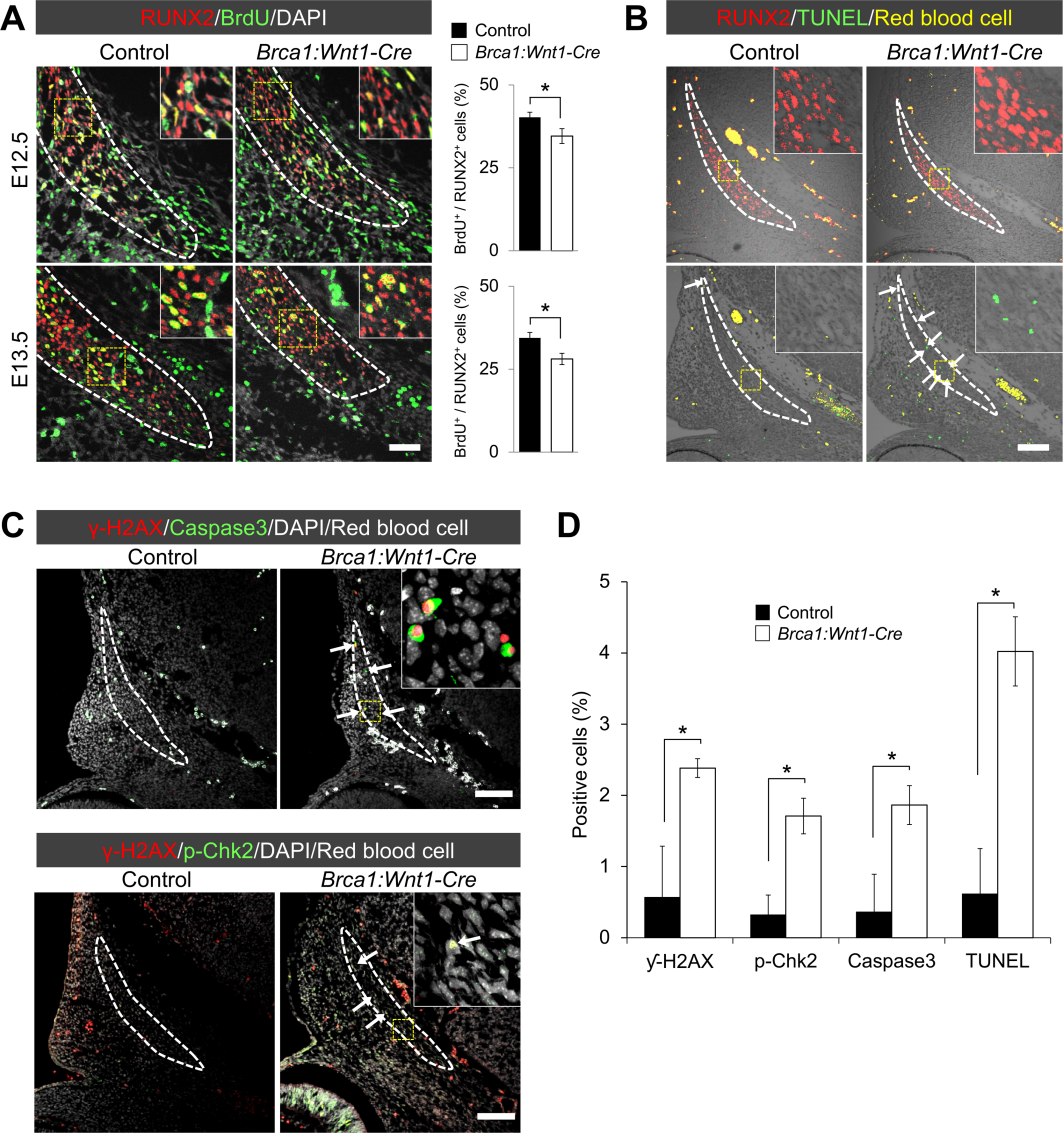
*Brca1* is indispensable for osteoblast proliferation and survival at mid-gestation. (A) Coronal sections of control and *Brca1*:*Wnt1-Cre* frontal bone primordia at E12.5 and E13.5 were double-labeled with RUNX2 (red) and BrdU (green) to detect osteogenic cells and proliferative cells, respectively. Broken line describes the osteogenic lineage cell population. Right charts show quantification of the ratio of BrdU-positive cells over RUNX2-positive cells. Scale bar = 50μm. (B) Immunostaining for RUNX2 and TUNEL assay of sections from control and *Brca1*:*Wnt1-Cre* embryos at E12.5. Broken line describes the osteogenic lineage cell population. White arrows indicate TUNEL-positive cells. Yellow color represents the non-specific signal from red blood cells. Scale bar = 100μm. (C) Immunostaining for γ-H2AX and Cleaved Caspase-3 and/or p-Chk2 of sections from control and *Brca1*:*Wnt1-Cre* embryos at E12.5. Broken line describes the osteogenic lineage cell population. White arrows indicate γ-H2AX- and/or Cleaved Caspase-3-positive cells. Scale bar = 100μm. (D) Quantification of the percentage of γ-H2AX-, Cleaved Caspase3- and TUNEL-positive cells in the frontal bone primordium. Data in A and D are represented as mean ±SD, n = 3 in each group. *P<0.05.

CNCC-derived osteogenic precursors migrate from the dorsal neural tube to the supraorbital ridge, and ectomesenchymal cells start to condense at embryonic day (E) 12.5 [17, 18]. Therefore, we asked whether the skull malformations in *Brca1*:*Wnt1-Cre* mice can be attributed to the lack of early osteogenic condensation at E12.5. To investigate the pathogenesis of skull defects in *Brca1*:*Wnt1-Cre* mice, we examined the expression of RUNX2, an early marker of osteogenic precursors [19, 20]. While osteogenic precursors were well induced in both control and *Brca1*:*Wnt1-Cre* mutants, osteogenic proliferation was reduced in *Brca1*:*Wnt1-Cre* skulls at E12.5 and E13.5 (Fig 2A). Additionally, a large amount of apoptotic cells was observed in *Brca1*:*Wnt1-Cre* skulls (Fig 2B, arrows). At late-gestation at E16.5, however, CNCC-derived osteoblasts in *Brca1*:*Wnt1-Cre* embryos showed normal cell proliferation and survival at levels comparable to the controls (S2 Fig). These results indicate that the defects seen in the frontal bones in *Brca1*:*Wnt1-Cre* embryos are due to the decreased osteogenic cell population in the frontal bone primordium.

Because cell death in premigratory CNCCs frequently leads to craniofacial bone abnormalities [21], we tested whether there is increased cell death in premigratory CNCCs in *Brca1*:*Wnt1-Cre* mutants. We performed TUNEL assay in control and *Brca1*:*Wnt1-Cre* mutant embryos at E8.5. As a result, no excess of apoptosis occurred in *Brca1* mutant embryos (S3A Fig), suggesting that *Brca1* does not play a major role in the survival of premigratory CNCCs. In addition, CNCC migration also appeared to occur normally in *Brca1*:*Wnt1-Cre* mutant embryos indicated by normal *Sox10* expression pattern at E9.5 (S3B Fig). Thus, BRCA1 is less likely to play a critical role in premigratory CNCCs. Interestingly, previous studies showed that *Brca1* is highly expressed only from mid- to late- gestations in the facial tissues [22, 23], suggesting that BRCA1 may become critical after post-migration of CNCCs for the onset of craniofacial bone development. We examined whether BRCA1 is produced in CNCC-derived skeletogenic precursors at E12.5, the stage when CNCC-derived osteogenic precursors start to form [17, 18]. Our results showed that BRCA1 was abundantly produced in craniofacial skeletogenic precursors in control embryos, while its production was completely abolished in *Brca1* mutants (S4 Fig). Together, these results demonstrate that BRCA1 is critical for the early onset of craniofacial skeletogenesis, but not for premigratory CNCCs.

### Increased DNA damage in *Brca1*:*Wnt1-Cre* embryos

It has been reported that DNA damage in neuroepithelial cells can induce cell death, leading to craniofacial bone deformities [24]. Because BRCA1 functions in DNA damage repair [12], one might predict that the underlying etiology of skull abnormalities is DNA damage-induced apoptosis in *Brca1*:*Wnt1-Cre* embryos. To address this hypothesis, we examined whether there is increased DNA damage in craniofacial skeletogenic precursors in *Brca1*:*Wnt1-Cre* embryos. We carried out immunofluorescence using antibodies against γ-H2AX, a marker of unrepaired DNA damage [25], in skulls from the embryos. Compared to the control, skulls from *Brca1*:*Wnt1-Cre* embryos showed increased numbers of γ-H2AX-stained skeletogenic precursor cells that are co-labeled with Cleaved Caspase-3 (Fig 2C, arrows). The portion of nuclei of skeletogenic precursor cells stained positive for phosphorylated-Chk2, a checkpoint kinase that is phosphorylated upon DNA damage and activation of central DNA damage kinase ATM, also increased (Fig 2C and 2D), indicating increased DNA damage-induced check point response. These data indicate that BRCA1 plays an important role in osteogenic proliferation and survival by suppressing DNA damage at the early stages of osteogenesis.

We also investigated whether BRCA1 plays a role in osteogenic differentiation. CNCC-derived skull tissues were harvested, and the expression levels of osteogenic genes were examined. The majority of the osteogenic genes tested, including *Runx2*, *Sp7* and *Ibsp*, were expressed normally in control and *Brca1*:*Wnt1-Cre* embryos (S5A Fig). Furthermore, *Brca1*-disrupted preosteoblasts from CNCC-derived skulls were capable to differentiate and mineralize *in vitro* (S5B Fig). Thus, *Brca1* is dispensable for osteogenic differentiation.

### Deletion of *p53* partially rescues the skull defects in *Brca1* mutant mice by decreasing DNA damage and preventing cell death

**Fig 3:**
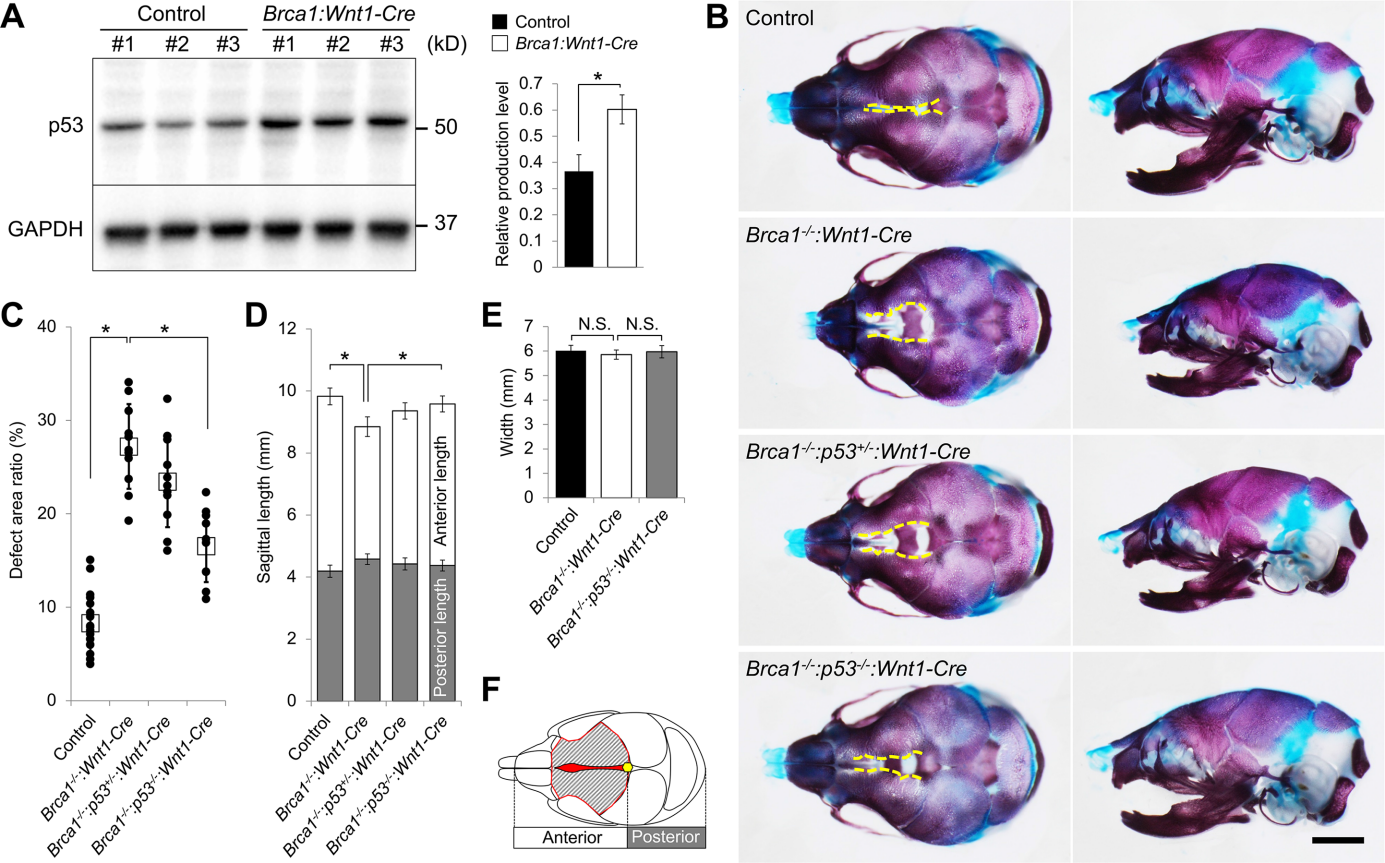
The *Brca1-p53* interaction is critical for craniofacial bone morphogenesis. (A) Immunoblot analysis of facial region tissue from E13.5 embryos. Each sample is from individual embryos. Right chart shows quantification of p53 relative production level. (B) Alcian blue- and alizarin red-stained skulls of control, *Brca1*^*-/-*^:*Wnt1-Cre*, *Brca1*^*-/-*^:*p53*^*+/-*^:*Wnt1-Cre* and *Brca1*^*-/-*^:*p53*^*-/-*^:*Wnt1-Cre* mice at birth. Yellow broken lines indicate osteogenic fronts. Scale bar = 2mm. (C) Quantification of the area ratio of frontal foramen in the frontal bone area. White box represents the mean of each genotype. (D) Quantification of sagittal length. (E) Quantification of skull width. (F) Measurement schema of C, D and E. The shaded area surrounded by the red line is the measured frontal area in C. Sagittal length was divided into anterior and posterior part at the estimated anterior fontanelle (yellow). Data in A, D and E are represented as mean ±SD, n = 3 in A and n = 10 in C, D and E in each group. *P<0.05. N.S., not significant.

It has been reported that DNA damage triggers p53 stabilization, and if the damage is extensive or cannot be repaired, it induces apoptosis [26]. Therefore, we hypothesized that p53-mediated apoptosis may cause the craniofacial bone defects seen in *Brca1*:*Wnt1-Cre* embryos. Supporting this hypothesis, the protein levels of p53 were much higher in *Brca1*:*Wnt1-Cre* mutants than in controls (Fig 3A), suggesting that DNA damage-induced p53 stabilization may be responsible for the pathogenesis of *Brca1*:*Wnt1-Cre* mice. To test this possibility *in vivo*, we superimposed null alleles of *p53* into *Brca1*:*Wnt1-Cre* mutants in a neural crest-specific manner [27]. Consistent with our prediction, the *p53* deletion partially rescued the craniofacial bone defects in *Brca1*:*Wnt1-Cre* mutants (Fig 3B). Importantly, two copies of the *p53* deletion alleles were able to rescue the skull defects more efficiently than one copy in *Brca1*:*Wnt1-Cre* embryos (Fig 3B and 3C). While there was no significant difference in head width in all genotypes, sagittal skull length was also rescued along with the recovery of maxilla and mandibular bones (Fig 3D–3F).

**Fig 4:**
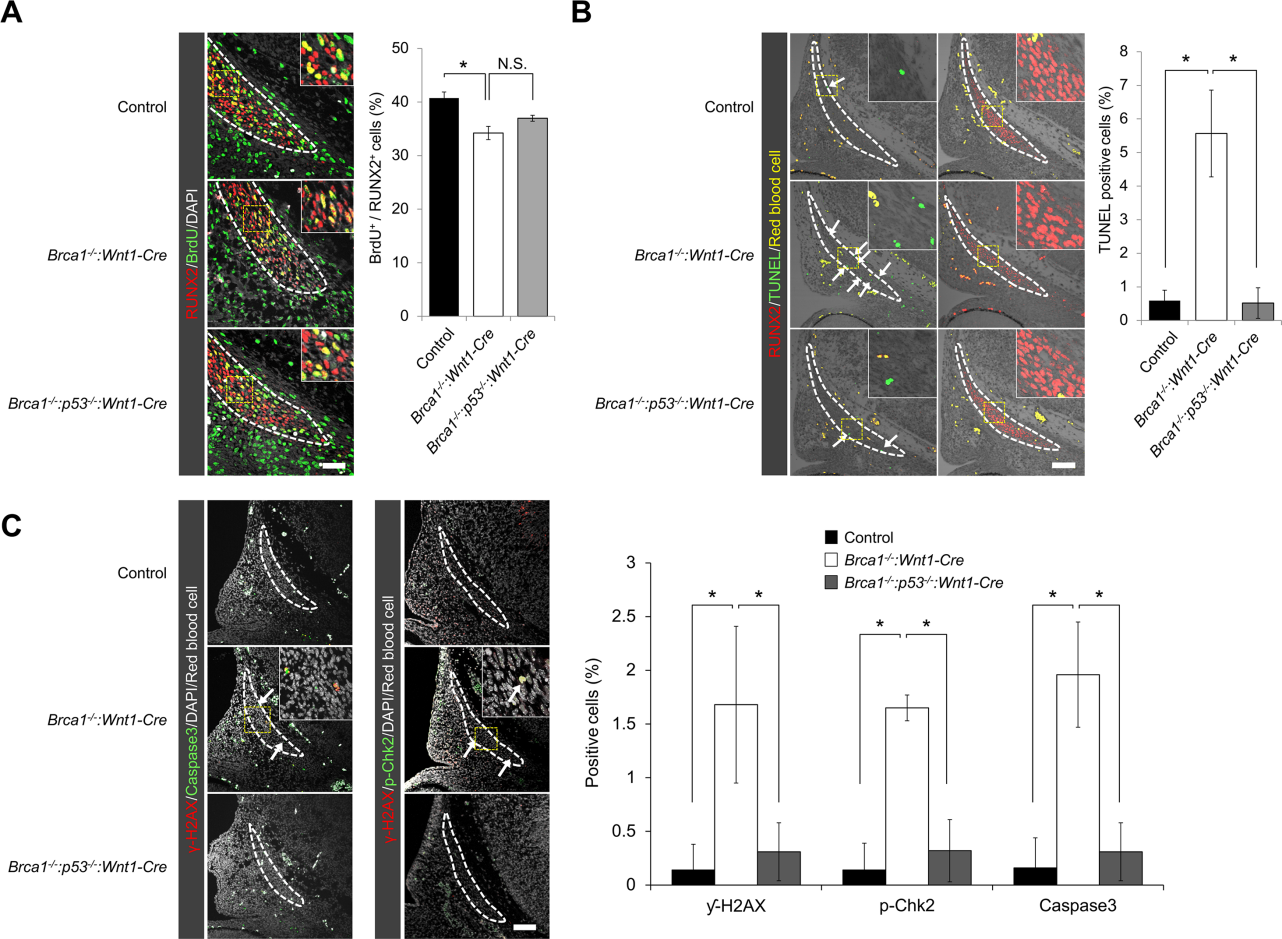
Deletion of *p53* partially rescues the skull defects by preventing cell death. (A) Coronal sections of control, *Brca1*^*-/-*^:*Wnt1-Cre* and *Brca1*^*-/-*^:*p53*^*-/-*^:*Wnt1-Cre* frontal bone primordia at E12.5 were double-labeled with RUNX2 (red) and BrdU (green). Broken line describes the osteogenic lineage cell population. Right charts show quantification of the ratio of BrdU-positive cells over RUNX2-positive cells. Scale bar = 50μm. (B) Immunostaining for RUNX2 and TUNEL assay of sections from control, *Brca1*^*-/-*^:*Wnt1-Cre* and *Brca1*^*-/-*^:*p53*^*-/-*^:*Wnt1-Cre* embryos at E12.5. Broken line describes the osteogenic lineage cell population. White arrows indicate TUNEL-positive cells. Yellow color represents the non-specific signal from red blood cells. Right chart shows quantification of the percentage of TUNEL-positive cells in the frontal bone primordium. (C) Immunostaining for γ-H2AX and Cleaved Caspase-3 and/or p-Chk2 of sections from control, *Brca1*^*-/-*^:*Wnt1-Cre* and *Brca1*^*-/-*^:*p53*^*-/-*^:*Wnt1-Cre* embryos at E12.5. Broken line describes the osteogenic lineage cell population. White arrows indicate double-positive cells for γ-H2AX/Cleaved Caspase-3 and/or γ-H2AX/p-Chk2. Right chart shows quantification of the percentage of γ-H2AX, Cleaved Caspase-3 and p-Chk2 positive cells in the frontal bone primordium. Scale bar = 100μm. Data in A, B and C are represented as mean ±SD, n = 3 in each group. *P<0.05. N.S., not significant.

We found that the *p53* deletion did not have an effect on the dysregulation of cell proliferation (Fig 4A), but sufficiently suppressed the enhanced cell death in *Brca1*:*Wnt1-Cre* embryos (Fig 4B). The suppression of cell death in rescued mice was linked to reduced number of γ-H2AX-stained cells co-labeled with either Cleaved Caspase-3 or phosphorylated-Chk2 (Fig 4C). Thus, reduction of *p53* in *Brca1*:*Wnt1-Cre* predominantly rescues the phenotype through decreasing DNA damage-induced cell death, but not through increasing osteogenic cell proliferation. We also examined whether p53 cell cycle regulation is involved in rescuing the defects of *Brca1*:*Wnt1-Cre* embryos. Since p53 regulates cell cycle through regulation of p21 [28], we analyzed p21 expression in *Brca1* mutants and rescued embryos. The expression levels of p21 were comparable among controls, *Brca1* mutants and *p53*^*-/-*^ superimposed *Brca1* mutants (S6 Fig). This indicates that p21 regulation was not affected and that the cell cycle checkpoint regulation through the p53-p21 axis did not play a major role in its function in *Brca1* mutants. Taken together, these data demonstrate that BRCA1 deficiency-induced pathogenesis of craniofacial bone defects can be partially rescued by inactivation of p53 through reducing DNA damage-induced cell death.

### BRCA2 is required for craniofacial skeletal development

BRCA2, the second major hereditary breast cancer susceptibility factor, is also critical for maintaining genome integrity [8]. While BRCA1 has multiple functions in the repair process of DNA damage, BRCA2 appears to be mainly involved in HR [29].

**Fig 5:**
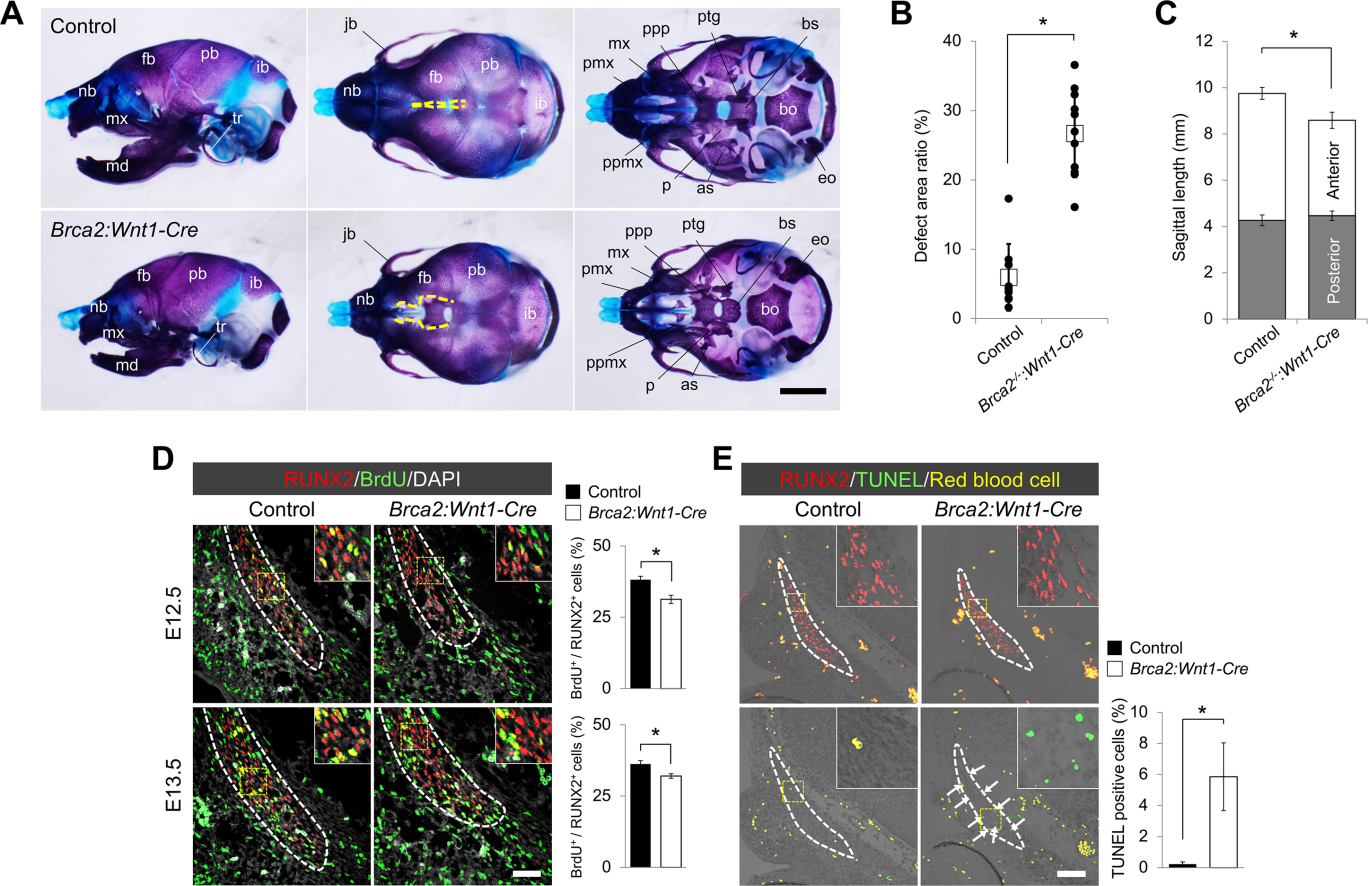
Neural crest cell-specific *Brca2* deletion results in a craniofacial bone phenotype similar to that of the neural crest cell-specific *Brca1* deletion in mice. (A) Alcian blue- and alizarin red-stained skulls of control and *Brca2*:*Wnt1-Cre* mice at birth. Yellow broken lines indicate osteogenic fronts. Scale bar = 2mm. (B) Quantification of the area ratio of frontal foramen in the frontal bone area. White box represents the mean of each genotype. (C) Quantification of sagittal length. (D) Coronal sections of control and *Brca2*:*Wnt1-Cre* frontal bone primordia at E12.5 and E13.5 were double-labeled with RUNX2 (red) and BrdU (green). Broken line describes the osteogenic lineage cell population. Right charts show quantification of the ratio of BrdU-positive cells over RUNX2-positive cells. Scale bar = 50μm. (E) Immunostaining for RUNX2 and TUNEL assay of sections from control and *Brca2*:*Wnt1-Cre* embryos at E12.5. Broken line describes the osteogenic lineage cell population. White arrows indicate TUNEL-positive cells. Yellow color represents the non-specific signal from red blood cells. Scale bar = 100μm. as, alisphenoid; bo, basioccipital; bs, basisphenoid; eo, exoccipital; fb, frontal bone; ib, interparietal bone; jb, jugal bone; md, mandible; mx, maxilla; nb, nasal bone; p, palatine; pb, parietal bone; pmx, premaxilla; ppmx, palatal process of maxilla; ppp, palatal process of palatine; ptg, pterygoid; tr, tympanic ring.

To examine the role of BRCA2 in CNCCs, we disrupted *Brca2* in an epithelial- and/or ectomesenchymal-cell specific manner in mice [30]. While the epithelial-specific deletion of *Brca2* did not induce any overt craniofacial malformation (S7 Fig), mice lacking *Brca2* in CNCCs (hereafter *Brca2*:*Wnt1-Cre*) exhibited craniofacial bone defects resembling those of *Brca1*:*Wnt1-Cre* mice (Fig 5A). CNCC-derived craniofacial bones, including the frontal-nasal and maxilla-mandibular bones, were severely compromised (Fig 5A). The ratio of defective frontal bone area and sagittal length in the skull was measured (Fig 5B and 5C). Similar to *Brca1*:*Wnt1-Cre* mice, CNCC-derived osteoblasts from *Brca2*:*Wnt1-Cre* embryos differentiated normally (S8 Fig). On the other hand, both osteogenic proliferation and cell survival were severely attenuated in *Brca2*:*Wnt1-Cre* (Fig 5D and 5E). With the strong phenotypic similarities in both *Brca1*:*Wnt1-Cre* and *Brca2*:*Wnt1-Cre* mutants, we conclude that BRCA1 and BRCA2 are critical for craniofacial skeletal development.

## Discussion

The etiology of craniofacial bone abnormalities is very complex. Alteration of transcriptional regulation, developmental signals, or epigenetic factors has been considered to cause facial bone defects. However, much still remains unknown about the regulation of craniofacial bone development. BRCA1 and BRCA2 breast tumor suppressor proteins play important roles in DNA damage response and repair, maintenance of genome stability and tumor suppression. In this report, we found a novel connection between the etiology of craniofacial skeletal defects and the BRCA1/BRCA2-dependent DNA damage response. Our data indicate that maintaining genomic stability through BRCA1/BRCA2 may be one of the key elements to prevent craniofacial bone abnormalities.

### The DNA damage repair machinery is critical for the onset of craniofacial bone development

BRCA1 has a central role in DNA damage response and tumor suppression [31], but its function in craniofacial bone development is not known. Conventional disruption of *Brca1* in mice results in embryonic lethality due to increased cell death and/or restricted proliferation in neuroepithelial cells [32–34], and mice lacking *Brca1* in neural stem cells display severe brain defects [35, 36]. These data indicate that BRCA1 may have a specific role in neuroepithelial cell proliferation and differentiation. Our findings support this notion that BRCA1 is critical for the neuroepithelial lineage cells, i.e. CNCC-derivatives. Our data showed that *Brca1* disruption in CNCCs causes decreased proliferation and increased cell death at mid gestation (Fig 2), but not at the maturation stages of craniofacial osteogenesis (S2 Fig). Thus, BRCA1 is likely required for early onset of craniofacial skeletogenesis.

Similar to our *Brca1*:*Wnt1-Cre* mice, treacle ribosome biogenesis factor 1 (*Tcof1*) haploinsufficiency leads to augmentation of p53 production, and genetic suppression of *p53* in *Tcof1* mutant mice rescues the craniofacial bone abnormalities [37]. Of note, *Tcof1* haploinsufficiency results in neuroepithelial cell death, and loss of *Tcof1* diminishes the accumulation of BRCA1 at DNA damage sites, providing a potential link between TCOF1 and BRCA1 in the DNA damage response [24]. It remains to be determined whether TCOF1 and BRCA1 genetically interact in craniofacial bone formation and whether TCOF1 is involved in the regulation of BRCA1.

### BRCA1/BRCA2-dependent DNA damage repair is essential for craniofacial bone development

Deficiencies in the DNA response and repair mechanism often lead to genome instability associated with cancer predisposition. Whereas the importance of BRCA1/BRCA2-dependent DNA damage response and repair in tumorigenesis is well known, it is unclear how the BRCA1/BRCA2-dependent pathway functions in multiple developmental aspects, especially during craniofacial formation. The main function of BRCA2 is to load Rad51 recombinase to single-stranded DNA to facilitate HR, while BRCA1 has been implicated in multiple aspects of cellular response in addition to its role in regulating HR. We found that *Brca2*:*Wnt1-Cre* mutants have craniofacial bone defects almost identical to those observed in *Brca1*:*Wnt1-Cre* mutants (Fig 1, Fig 2 and Fig 5), suggesting that BRCA1- and BRCA2-dependent HR function is likely to be involved in regulating craniofacial bone development. It is possible that CNCCs encounter internal or external genetic insults that can result in DNA damage in early embryonic development during craniofacial bone formation. Indeed, it has been shown that oxidative stress increases during this time of the development process, which may lead to increased DNA damage [24]. Therefore, BRCA1/BRCA2-dependent HR is likely to be essential to ensure proper CNCC proliferation. Recently, it also has been shown that BRCA1/BRCA2 play an important role in protecting stalled replication fork stability in order to maintain genome integrity [12]. It is also possible that rapid CNCC proliferation during craniofacial bone formation may generate replication stress; BRCA1/BRCA2 are required to protect the replication fork's integrity, and their deficiency leads to increased DNA damage. Given the essential role of BRCA1 and BRCA2 in embryonic development, the *Wnt1-Cre* deletion of *Brca1* or *Brca2* resulted in relatively mild craniofacial phenotypes. It remains to be seen whether BRCA1 and BRCA2 function redundantly during neural crest cell development and their differentiation into skeletogenic derivatives. Generating a *Brca1*- and *Brca2*-double deficiency in CNCCs may facilitate the understanding of the role of BRCA1 and BRCA2 in craniofacial bone development. Nevertheless, although the importance of BRCA1/BRCA2-dependent function in tumorigenesis is widely recognized, our data show, for the first time, the robust requirement of BRCA1 and BRCA2 in craniofacial bone development.

In summary, we demonstrate the function of BRCA1 and BRCA2 in CNCCs and highlight the importance of BRCA1/BRCA2-dependent function in DNA damage response and repair for proper craniofacial bone development.

## Materials and methods

### Ethics statement

The guidelines for this study were issued by the Center for Laboratory Animal Medicine and Care (CLAMC). The experimental protocol was reviewed and approved by the Animal Welfare Committee and the Institutional Animal Care and Use Committee of The University of Texas Medical School at Houston under approval number AWC-15-0152.

### Animals

*Brca1*-floxed mice [14], *Brca2*-floxed mice [30], *p53*-floxed mice [27], *Wnt1-Cre* mice [15], and *K14-Cre* mice [16] were obtained from NCI/NIH and The Jackson Laboratory. All mice were maintained in the animal facility of The University of Texas Medical School at Houston. The experimental protocol was reviewed and approved by the Animal Welfare Committee and the Institutional Animal Care and Use Committee of The University of Texas Medical School at Houston.

### Genotyping analysis

The DNA from mouse embryos was analyzed by PCR. The sequences of each primers were listed (S2 Table). The conditions for genotyping PCR were 94°C for 20 sec, 65°C for 20 sec, 72°C for 20 sec, repeated 40 cycles. Genotyping for *Brca1*, PCR reaction yielded 450 bp for wild-type or 500 bp for *Brca1* floxed DNA fragment. Genotyping for *Brca2*, PCR reaction yielded 298 bp for wild-type or 376 bp for *Brca2* floxed DNA fragment. Genotyping for *p53*, PCR reaction yielded 270 bp for wild-type or 390 bp for *p53* floxed DNA fragment. For genotyping of Cre, PCR reaction yielded 169 bp for Cre gene.

### Skeletal preparations and histological analysis

Staining of bone and cartilage of embryos with Alizarin red/Alcian blue was carried out as described previously [38]. An Olympus SZX16 microscope equipped with a DP71 digital camera was used for capturing images. The length and area ratio of the frontal foramina were measured by ImageJ. Immunofluorescent staining, and TUNEL assays of paraffin sections were performed as previously described [38]. Pregnant females were injected intraperitoneally with BrdU (Invitrogen, BrdU labeling reagent; 1 mL/100 g body weight) and sacrificed 2 hours later. Primary antibodies used in immunofluorescence staining were as follows: RUNX2 (1:400, Cell Signaling; no. 12556), Ki-67 (1:100, BD Biosciences; 550609), Cleaved Caspase-3 (1:400, Cell Signaling; 9664), γ-H2AX (1:200, Millipore; 05–636). Slides were viewed with an Olympus FluoView FV1000 laser scanning confocal microscope using the software FV10-ASW Viewer (version 3.1).

### Isolation of primary osteoblasts

Cranial preosteoblasts were established from E18.5 embryos as described previously [38]. The frontal bones were subjected to five sequential digestions with an enzyme mixture containing 1 mg/mL collagenase type I (Sigma-Aldrich; C0130) and 1 mg/mL collagenase type II (Sigma-Aldrich; C6885). Cell fractions (from two to five of the sequential digestions) were collected. Preosteoblasts were grown in α-MEM medium (Sigma-Aldrich; M8042) supplemented with 10% (vol/vol) FBS (Sigma-Aldrich; F4135), 100 U/mL penicillin-streptomycin (Sigma-Aldrich; P4333), and 4 mM glutamine (Sigma-Aldrich; G7513). Osteogenic differentiation was induced using culture medium supplemented with 50 μg/mL ascorbic acid (Sigma-Aldrich; A-4403) and 2 mM β-glycerophosphate (Sigma-Aldrich; G-9891).

### Western blot analysis

Facial tissues were homogenized using protein lysis buffers. After centrifugation, the supernatants were separated by SDS/PAGE, blotted onto a PVDF membrane, and analyzed with specific antibodies. The antibodies used were as follows: GAPDH (1:2000, Cell Signaling; 2118), p53 (1:2000, Santa Cruz; sc-6243). The Clarity Max ECL Substrate (Bio-rad) was used for chemiluminescent detection, and signals were quantified with Image Lab Version 5.0 (Bio-Rad).

### Quantitative RT-PCR

Using TRIzol Reagent (Thermo Fisher Scientific), total RNA was extracted from the frontal bones of control, *Brca1*:*Wnt1-Cre* and *Brca2*:*Wnt1-Cre* embryos at E17.5. The total RNA was treated with DNase I (Roche) before cDNA synthesis. Total RNA from the cultured osteoblasts was purified using the RNeasy Plus Mini Kit (Qiagen). cDNA was synthesized using iScript Reverse Transcription Supermix for RT-qPCR (BioRad). Quantitative RT-PCR was carried out using SsoAdvanced Universal SYBR Green Supermix (Bio-Rad) using CFX Connect System (Bio-Rad). The conditions for qRT-PCR were 95°C for 2 min, 95°C for 5 sec, 60°C for 30 sec, repeated 40 cycles. The sequences of each primers were listed below (Table 1). Data were normalized to GAPDH and quantified by 2^−∆∆CT^ method.

**Table 1:** Primer sequences for Quantitative RT-PCR analysis.

Gene Name	Forward primer 5’→3’	Reverse primer 5’→3’
*Runx2*	TATGGCGTCAAACAGCCTCT	GCTCACGTCGCTCATCTTG
*Sp7*	CTCTCCATCTGCCTGACTCC	CCAAATTTGCTGCAGGCT
*Ibsp*	GTCTTTAAGTACCGGCCACG	TGAAGAGTCACTGCCTCCCT
*Bglap*	AAGCAGGAGGGCAATAAGGT	CAAGCAGGGTTAAGCTCACA
*Fgfr1*	TCACAGCCACTCTCTGCACT	GTGGACCAGGAGAGACTCCA
*Fgfr2*	TCCATCAACCACACCTACCA	TGCAGACAAACTCCACATCC
*Fgfr3*	CTCTGGAGCCATGGTAGTCC	CTCCTGCTGGCTAGGTTCAG
*Gapdh*	CGTCCCGTAGACAAAATGGT	TCAATGAAGGGGTCGTTGAT

### Statistical analysis

The Student’s *t* test was used for statistical analysis. A *P* value of less than 0.05 was considered statistically significant.

## Supporting information

S1 Fig Alcian blue- and alizarin red-stained skulls of control and *Brca1*:*K14-Cre* mice at birth. Scale bar = 2mm. bo, basioccipital; bs, basisphenoid; fb, frontal bone; ib, interparietal bone; jb, jugal bone; md, mandible; mx, maxilla; nb, nasal bone; p, palatine; pb, parietal bone; ppp, palatal process of palatine; ptg, pterygoid; tr, tympanic ring.
(TIF)

S2 Fig (A) Double immunostaining for RUNX2 (red) and Ki-67 (green) of sections from control and *Brca1*:*Wnt1-Cre* embryos at E16.5. Broken line describes the osteogenic lineage cell population. Right chart show quantification of the ratio of Ki-67-positive cells over RUNX2-positive cells. Scale bar = 100μm. (B) TUNEL assay of sections from control and *Brca1*:*Wnt1-Cre* embryos at E16.5. Broken line describes the osteogenic lineage cell population. White arrows indicate TUNEL-positive cells. Right chart show quantification of the percentage of TUNEL-positive cells in the frontal bone primordium. Scale bar = 100μm. Data in A and B are represented as mean ±SD, n = 3 in each group. N.S., not significant.
(TIF)

S3 Fig (A) Whole mount TUNEL assay in control and *Brca1*:*Wnt1-Cre* embryos at E8.5. Scale bar = 100μm. (B) Expression analysis of *Sox10* by whole mount *in situ* hybridization in control and *Brca1*:*Wnt1-Cre* embryos at E9.5. Scale bar = 1mm in left panel and 250μm in right panel.
(TIF)

S4 Fig Immunostaining for BRCA1 (green) and RUNX2 (red) of sections from control and *Brca1*:*Wnt1-Cre* embryos at E12.5. Broken line describes the osteogenic lineage cell population. Scale bar = 100μm.
(TIF)

S5 Fig (A) Quantitative RT-PCR analysis of osteogenic markers in control and *Brca1*:*Wnt1-Cre* frontal bones at E17.5. (B) Bone nodules are stained with Alizarin red for primary osteoblasts derived from frontal bone culturing 1 week (1W) and 2 weeks (2W) and quantified (n = 3). Data in A and B are represented as mean ±SD, n = 3 in each group. N.S., not significant.
(TIF)

S6 Fig Immunostaining for p21 (green) and RUNX2 (red) of sections from control and *Brca1*:*Wnt1-Cre* and *Brca1*^*-/-*^:*p53*^*-/-*^:*Wnt1-Cre* embryos at E12.5. Broken line describes the osteogenic lineage cell population. Dental tissues served as positive controls for p21 since p21 was highly produced in the dental epithelium (green, right panel). Scale bar = 100 μm for skull tissues, 20 μm for dental tissue.
(TIF)

S7 Fig Alcian blue- and alizarin red-stained skulls of control and *Brca2*:*K14-Cre* mice at birth. Scale bar = 2mm. as, alisphenoid; bo, basioccipital; bs, basisphenoid; fb, frontal bone; ib, interparietal bone; jb, jugal bone; md, mandible; mx, maxilla; nb, nasal bone; p, palatine; pb, parietal bone; pmx, premaxilla; ppmx, palatal process of maxilla; ppp, palatal process of palatine; ptg, pterygoid; tr, tympanic ring.
(TIF)

S8 Fig Quantitative RT-PCR analysis of osteogenic markers in control and *Brca2*:*Wnt1-Cre* frontal bones at E17.5. (TIF)

S1 Table Genotype analysis of *Brca1* and *Brca2* mutant mice. *Brca1*:*Wnt1-Cre* mice and *Brca2*:*Wnt1-Cre* mice were born at Mendelian ratios but they could not survive more than twenty-four hours.
(TIF)

S2 Table Genotype analysis of *Brca1*, *Brca2* and *p53* mutant mice. The sequences of each primers were listed.
(TIF)
