# Supplementary figures and images for: BRCA1 and BRCA2 tumor suppressors in neural crest cells are essential for craniofacial bone development
Source: PLoS Genet. 2018 May 2;14(5):e1007340. doi: 10.1371/journal.pgen.1007340 (PMC5951594; doi:10.1371/journal.pgen.1007340)

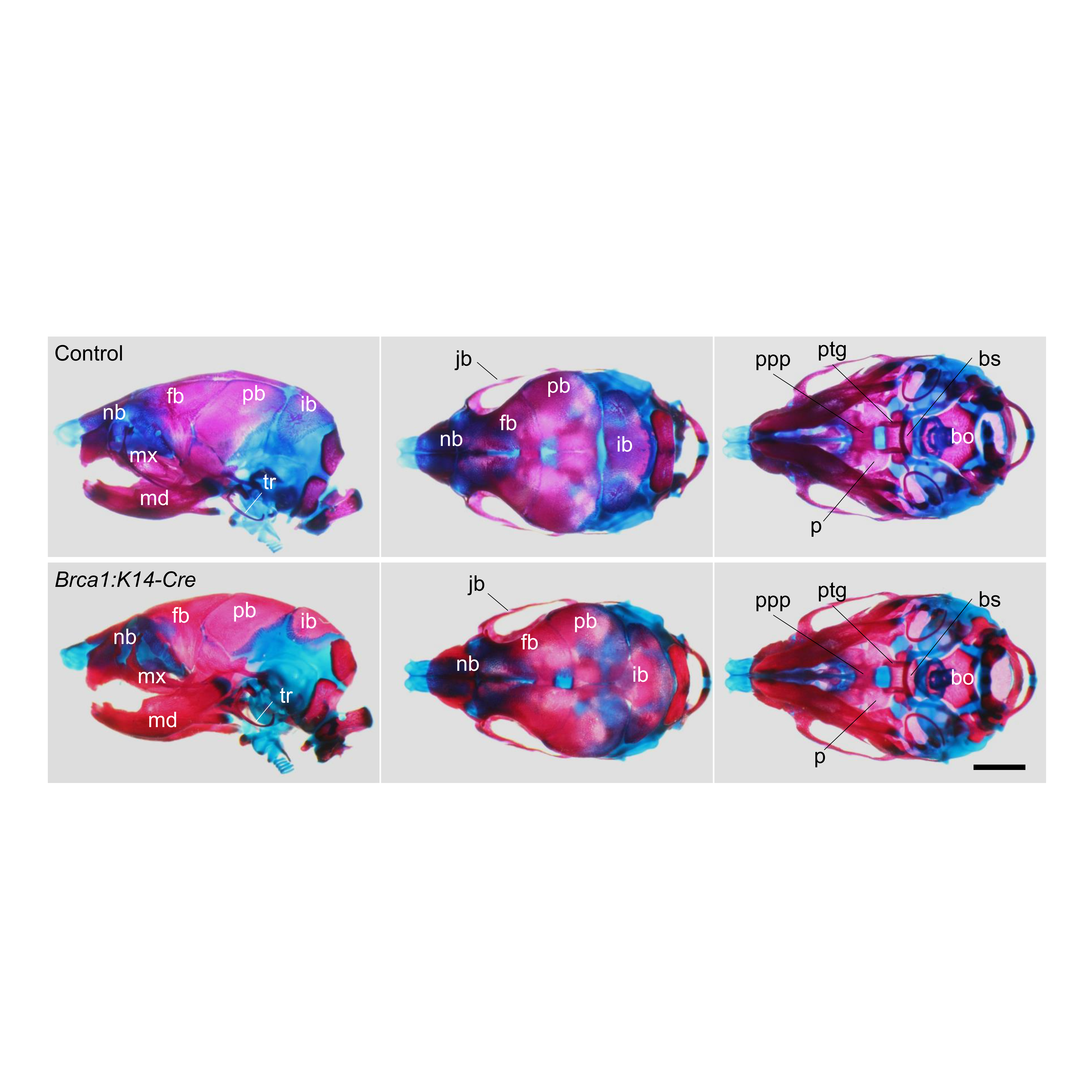

Supplement: S1 Fig — Scale bar = 2mm. bo, basioccipital; bs, basisphenoid; fb, frontal bone; ib, interparietal bone; jb, jugal bone; md, mandible; mx, maxilla; nb, nasal bone; p, palatine; pb, parietal bone; ppp, palatal process of palatine; ptg, pterygoid; tr, tympanic ring. (TIF) [file pgen.1007340.s001.tif]

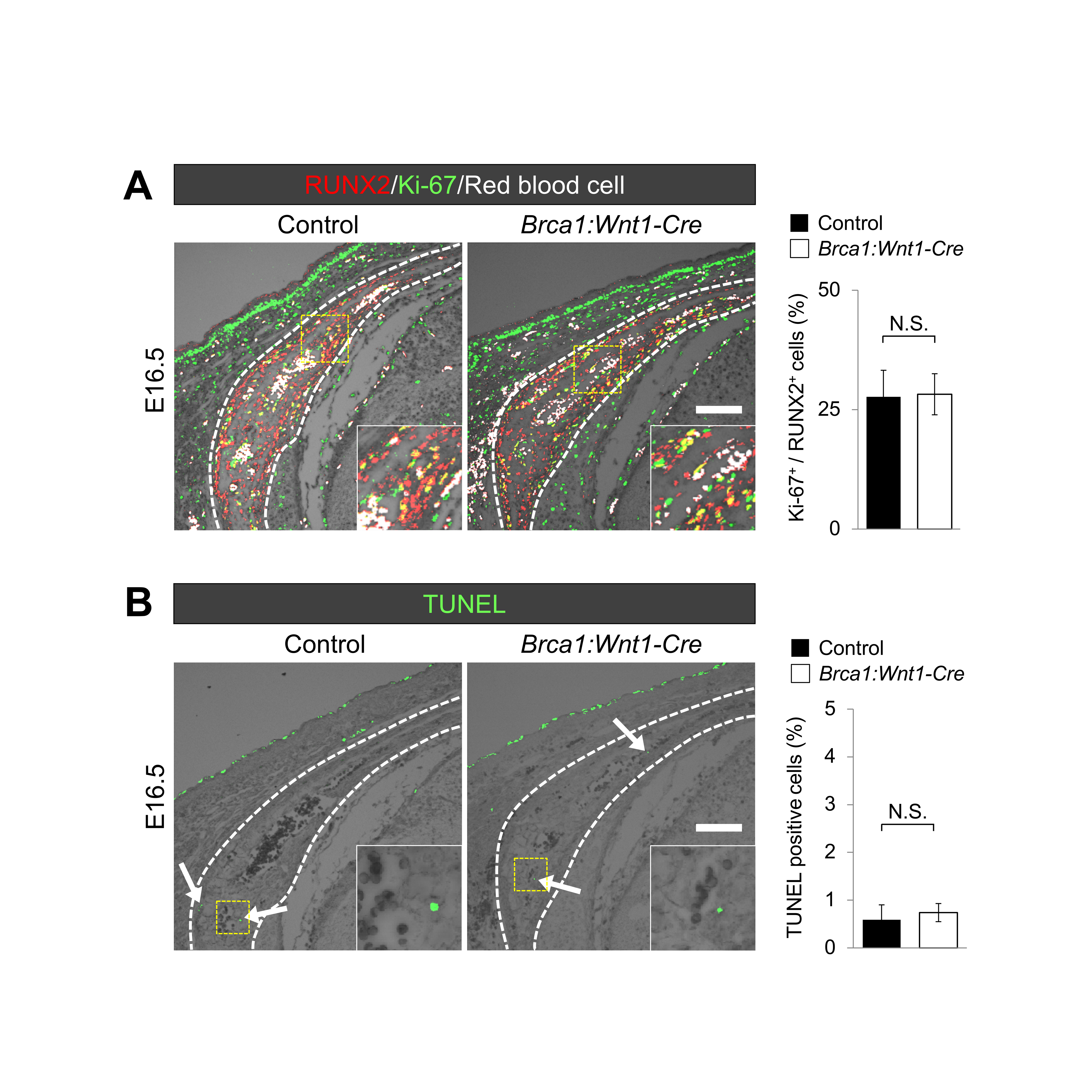

Supplement: S2 Fig — (A) Double immunostaining for RUNX2 (red) and Ki-67 (green) of sections from control and Brca1:Wnt1-Cre embryos at E16.5. Broken line describes the osteogenic lineage cell population. Right chart show quantification of the ratio of Ki-67-positive cells over RUNX2-positive cells. Scale bar = 100μm. (B) TUNEL assay of sections from control and Brca1:Wnt1-Cre embryos at E16.5. Broken line describes the osteogenic lineage cell population. White arrows indicate TUNEL-positive cells. Right chart show quantification of the percentage of TUNEL-positive cells in the frontal bone primordium. Scale bar = 100μm. Data in A and B are represented as mean ±SD, n = 3 in each group. N.S., not significant. (TIF) [file pgen.1007340.s002.tif]

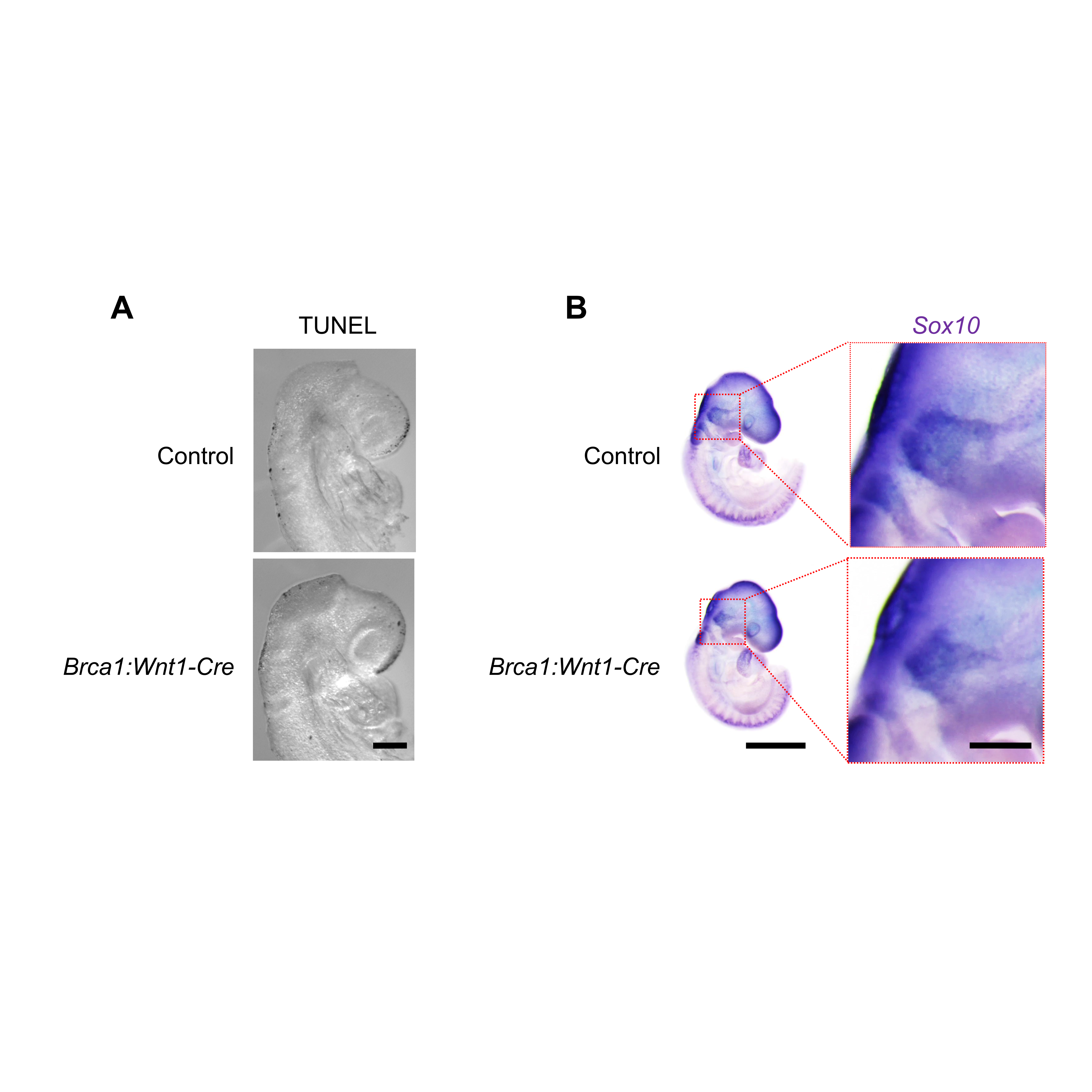

Supplement: S3 Fig — (A) Whole mount TUNEL assay in control and Brca1:Wnt1-Cre embryos at E8.5. Scale bar = 100μm. (B) Expression analysis of Sox10 by whole mount in situ hybridization in control and Brca1:Wnt1-Cre embryos at E9.5. Scale bar = 1mm in left panel and 250μm in right panel. (TIF) [file pgen.1007340.s003.tif]

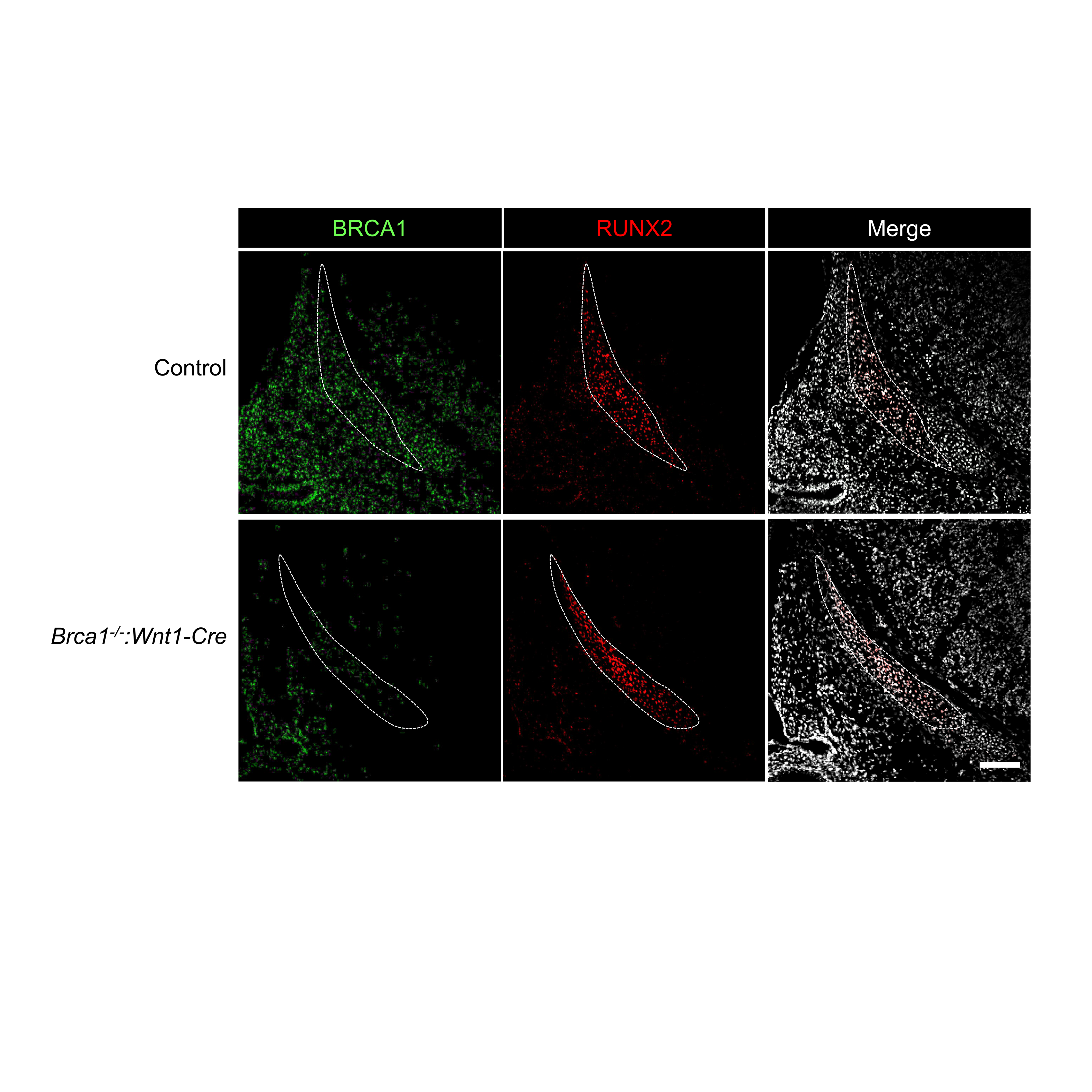

Supplement: S4 Fig — Broken line describes the osteogenic lineage cell population. Scale bar = 100μm. (TIF) [file pgen.1007340.s004.tif]

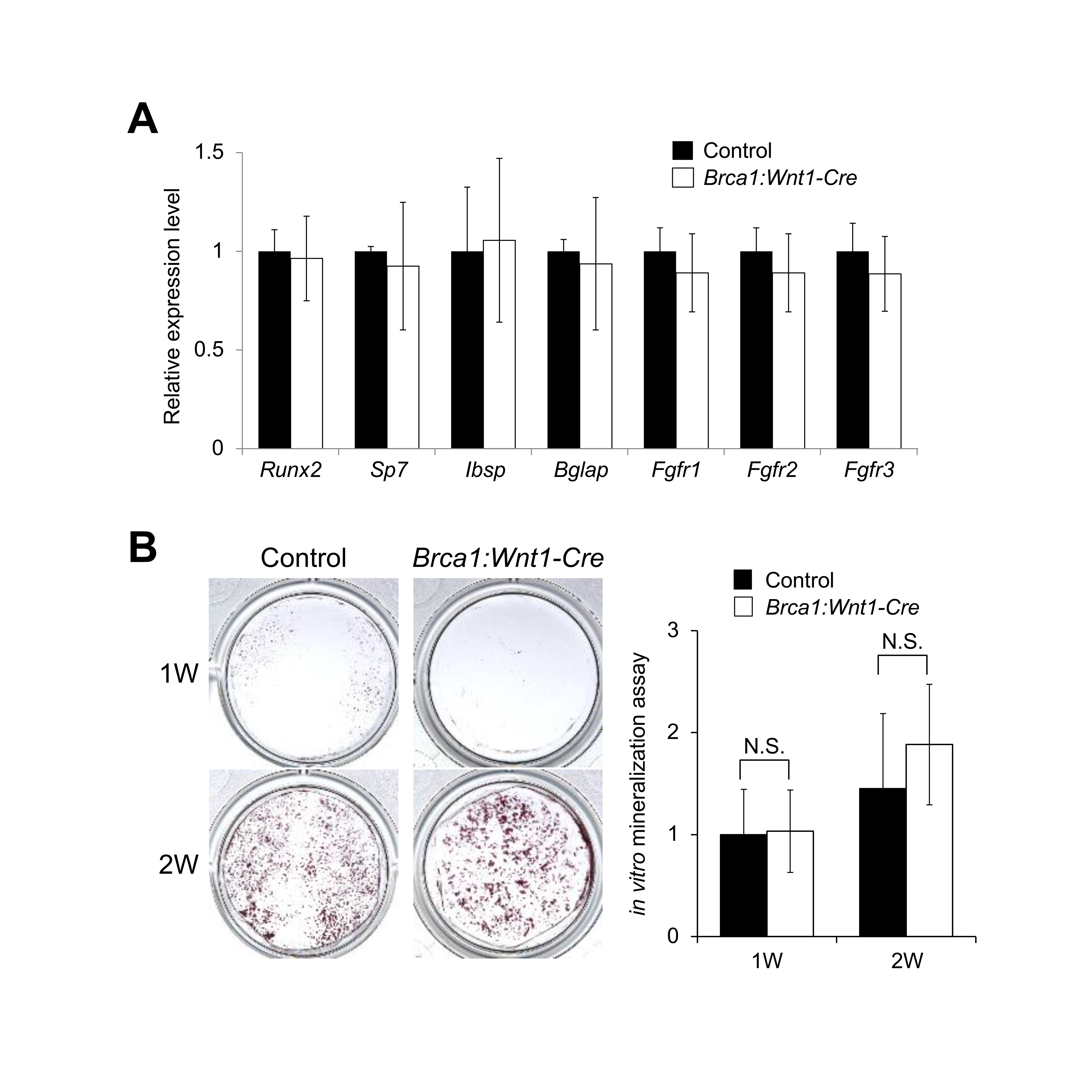

Supplement: S5 Fig — (A) Quantitative RT-PCR analysis of osteogenic markers in control and Brca1:Wnt1-Cre frontal bones at E17.5. (B) Bone nodules are stained with Alizarin red for primary osteoblasts derived from frontal bone culturing 1 week (1W) and 2 weeks (2W) and quantified (n = 3). Data in A and B are represented as mean ±SD, n = 3 in each group. N.S., not significant. (TIF) [file pgen.1007340.s005.tif]

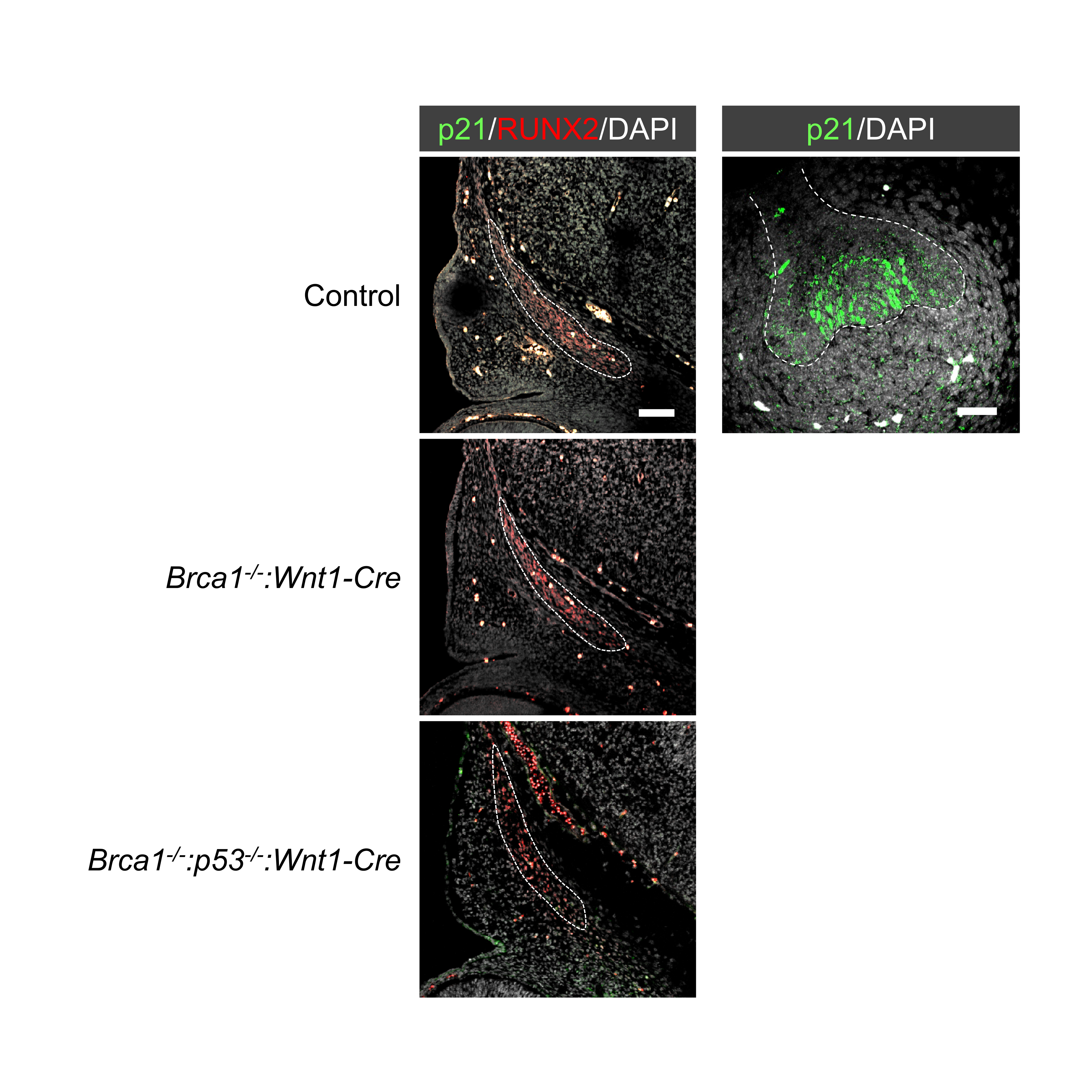

Supplement: S6 Fig — Broken line describes the osteogenic lineage cell population. Dental tissues served as positive controls for p21 since p21 was highly produced in the dental epithelium (green, right panel). Scale bar = 100 μm for skull tissues, 20 μm for dental tissue. (TIF) [file pgen.1007340.s006.tif]

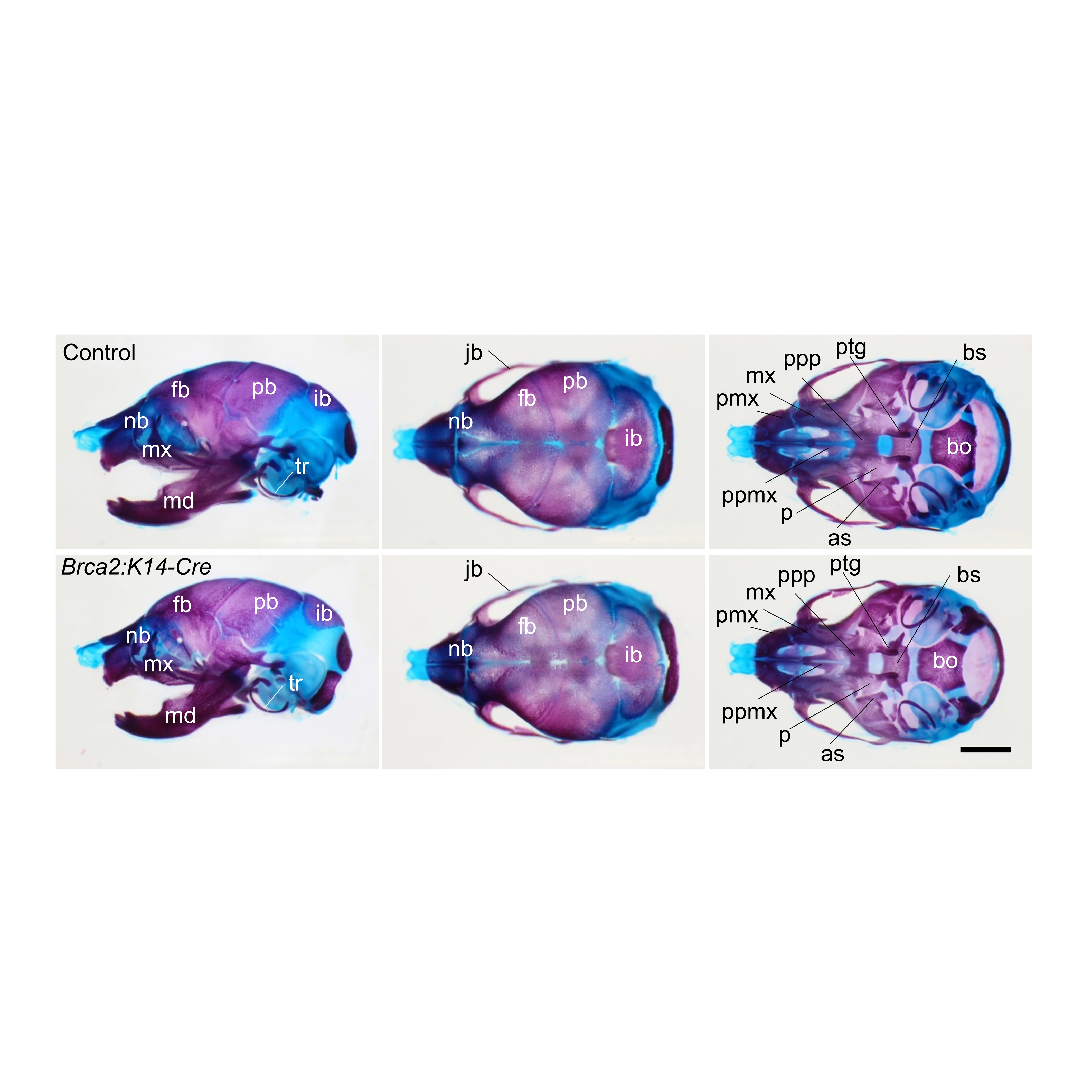

Supplement: S7 Fig — Scale bar = 2mm. as, alisphenoid; bo, basioccipital; bs, basisphenoid; fb, frontal bone; ib, interparietal bone; jb, jugal bone; md, mandible; mx, maxilla; nb, nasal bone; p, palatine; pb, parietal bone; pmx, premaxilla; ppmx, palatal process of maxilla; ppp, palatal process of palatine; ptg, pterygoid; tr, tympanic ring. (TIF) [file pgen.1007340.s007.tif]

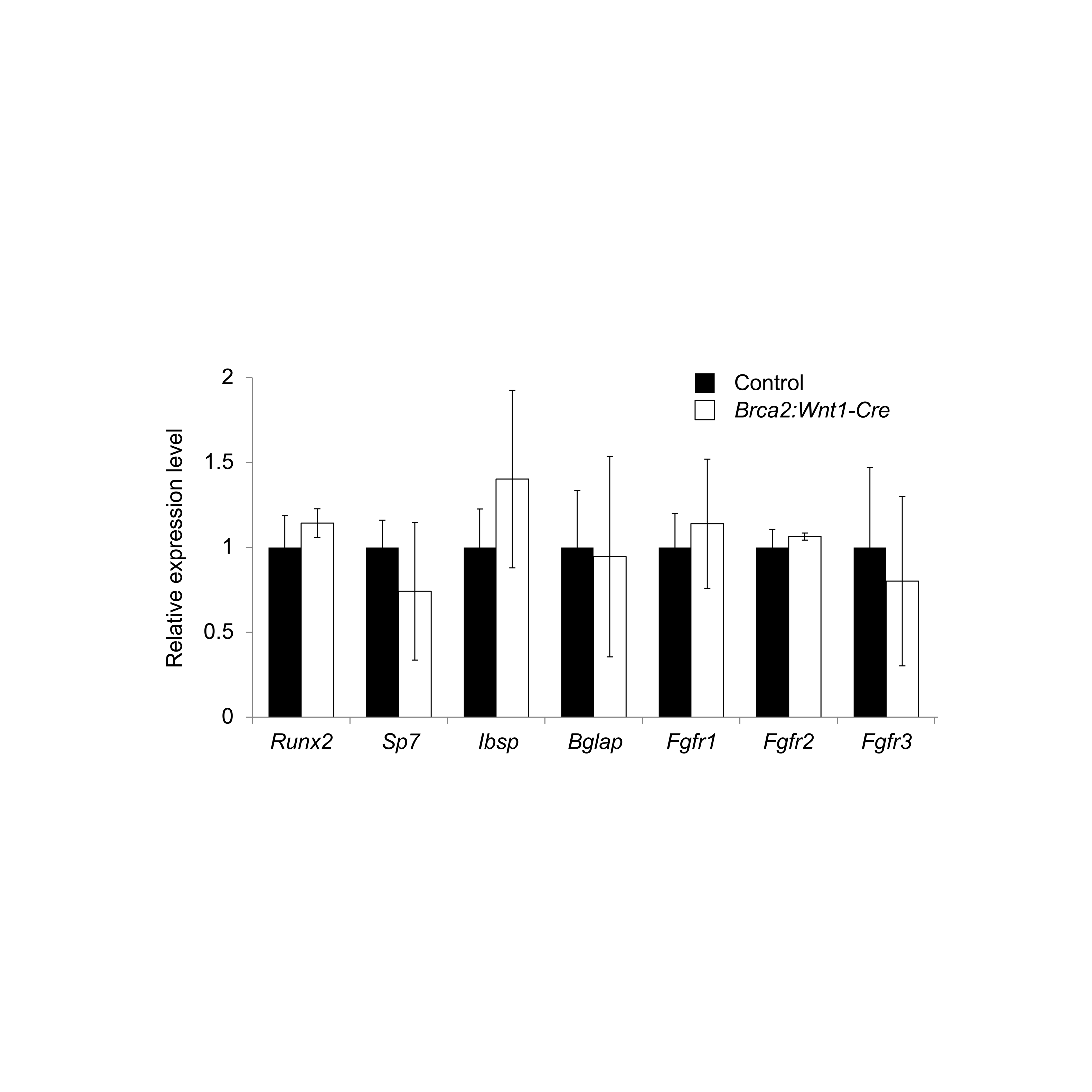

Supplement: S8 Fig — (TIF) [file pgen.1007340.s008.tif]

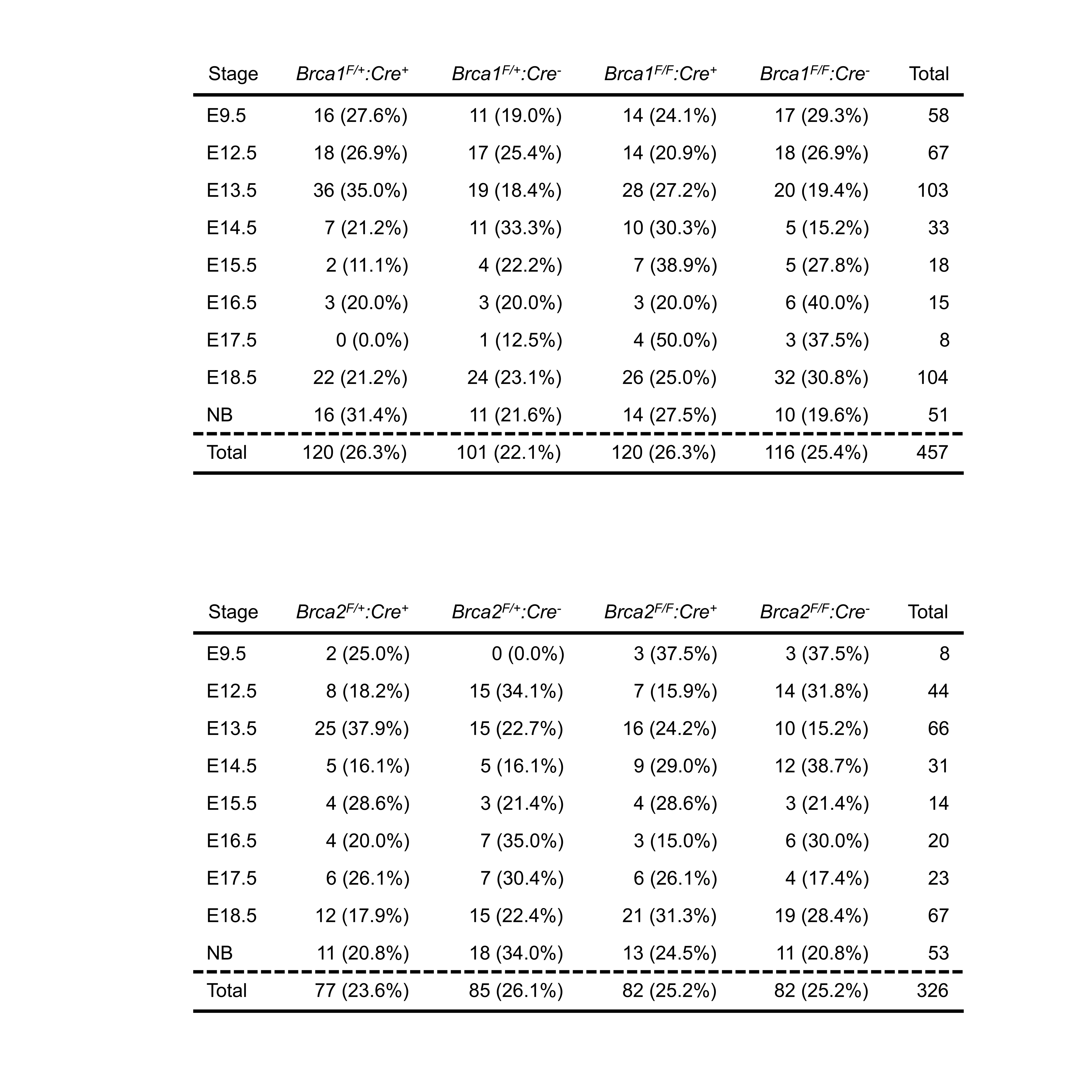

Supplement: S1 Table — Brca1:Wnt1-Cre mice and Brca2:Wnt1-Cre mice were born at Mendelian ratios but they could not survive more than twenty-four hours. (TIF) [file pgen.1007340.s009.tif]

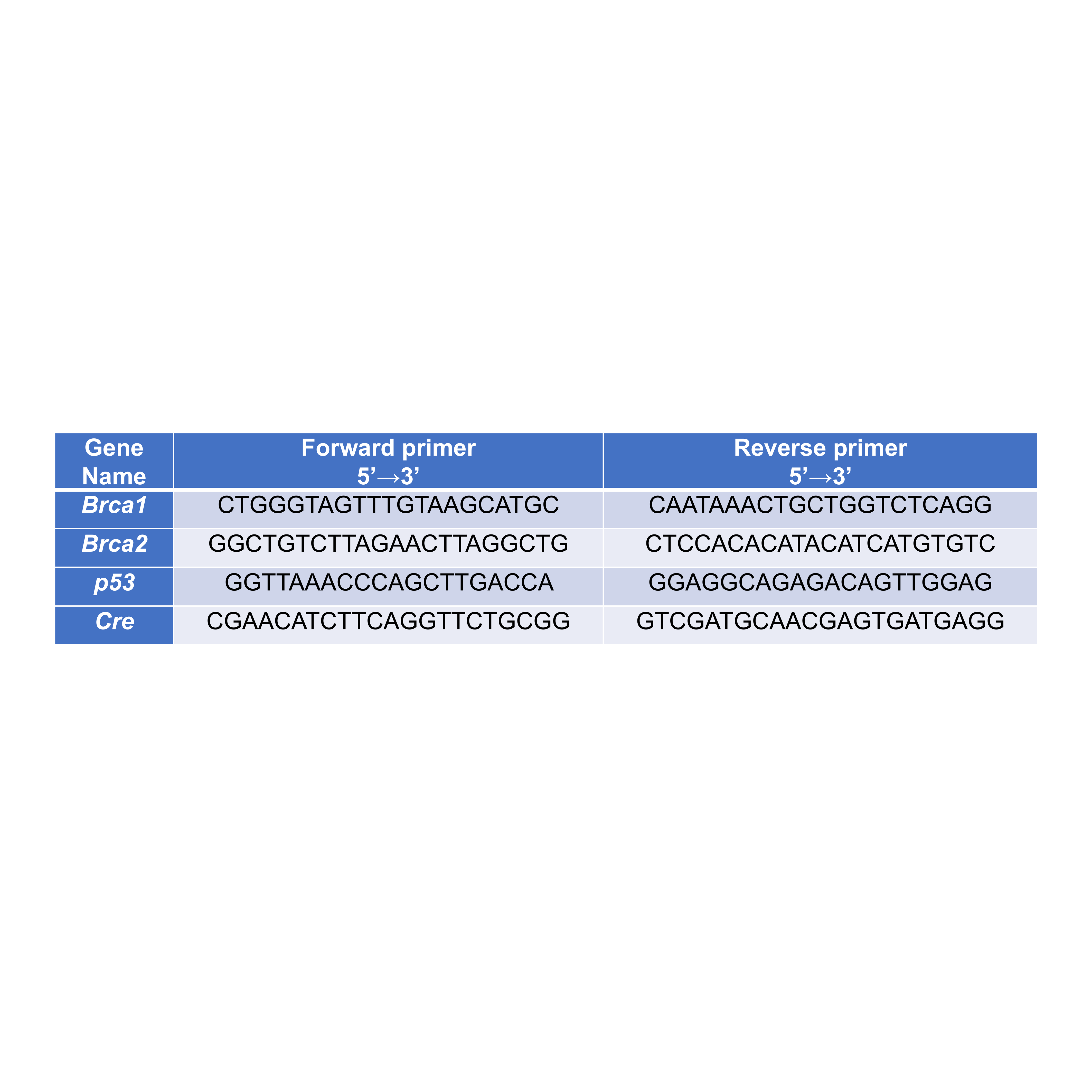

Supplement: S2 Table — The sequences of each primers were listed. (TIF) [file pgen.1007340.s010.tif]
